# 401. Host, treatment, and disease-related risk factors for invasive fungal disease in 549 patients with newly diagnosed high-risk myeloid malignancies treated with intensive induction chemotherapy

**DOI:** 10.1093/ofid/ofaf695.139

**Published:** 2026-01-11

**Authors:** Victor Kovac, Archana Sasi, Jessica S Little, Yiwen Liu, Ann E Woolley, Marlise Luskin, Shai Shimony, R Coleman Lindsley, Lindsey R Baden, Rahul S Vedula

**Affiliations:** Brigham and Women's Hospital, Boston, MA; Dana Farber Cancer Institute, Boston, Massachusetts; Brigham and Women's Hospital, Boston, MA; Dana Farber Cancer Institute, Boston, Massachusetts; Brigham and Women's Hospital, Boston, MA; Dana-Farber Cancer Institute, Boston, Massachusetts; Dana Farber Cancer Institute, Boston, Massachusetts; Dana Farber Cancer Institute, Boston, Massachusetts; Brigham and Women's Hospital, Boston, MA; Dana Farber Cancer Institute, Boston, Massachusetts

## Abstract

**Background:**

Patients with acute myeloid leukemia are at the greatest risk of invasive fungal disease (IFD) of any malignancy, prompting recommendations for antifungal prophylaxis with intensive induction chemotherapy. However, this practice is not adopted at our center, permitting an unbiased assessment of host, treatment, and disease-related risk factors for IFD.

**Table 1.**
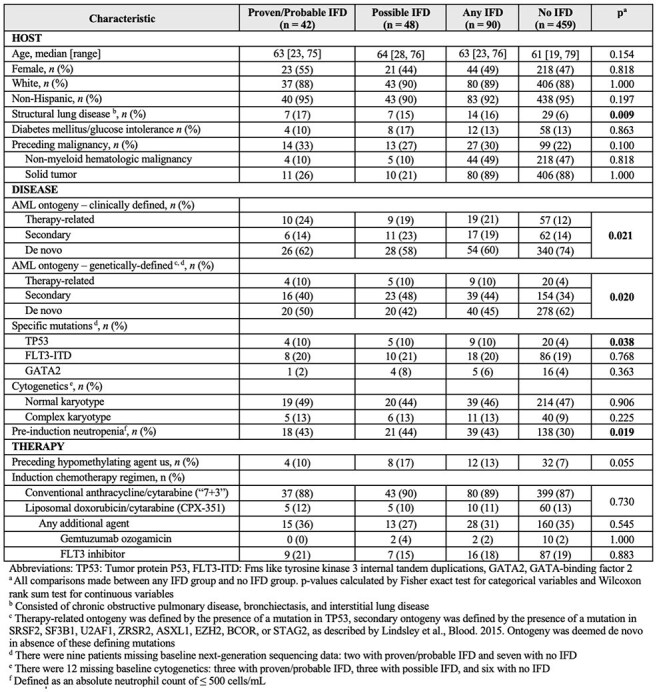
Baseline Characteristics of 549 Patients with High-Risk Myeloid Malignancies Treated with Intensive Induction Chemotherapy, Stratified by Invasive Fungal Disease Status in the First 100 Days

**Table 2.**
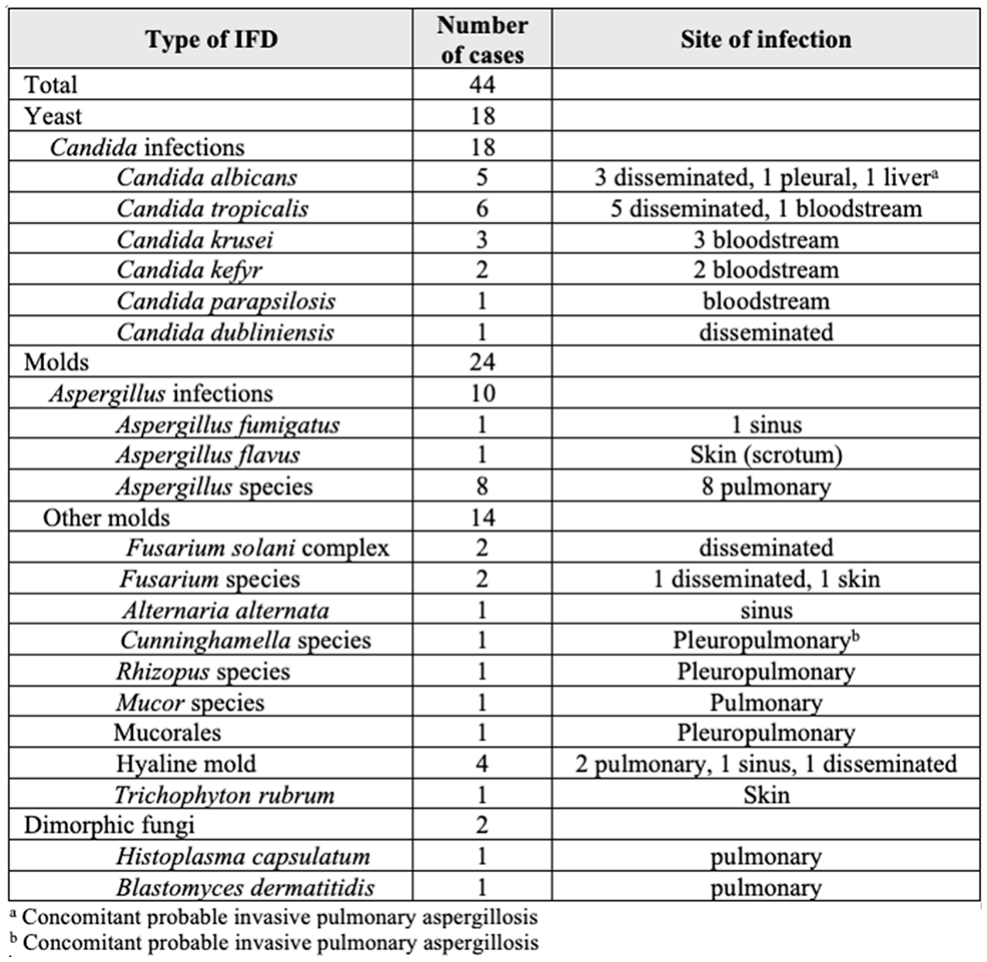
Proven and Probable Invasive Fungal Disease in the First 100 Days of Intensive Induction

Chemotherapy for High-Risk Myeloid Malignancies

**Methods:**

We performed a retrospective analysis of patients with newly diagnosed high-risk myeloid malignancies (MM) treated with intensive induction chemotherapy at Dana-Farber Cancer Center from July 2014 to December 2023. We compared demographic, clinical, and AML-related data stratified by the primary outcome of IFD within 100 days following induction. IFD was classified by EORTC/MSG 2020 criteria and adjudicated by two infectious diseases physicians.

**Results:**

We identified 549 consecutive patients treated with intensive induction for newly diagnosed MM at our institution. In total, 42 patients (7.6%) developed proven or probable IFD, 48 (8.7%) developed possible IFD, and 459 (84%) did not develop IFD (Table 1). There were 44 proven/probable IFD cases in 42 individual patients of which 24 were molds (54%) and 18 were yeast (41%) (Table 2). Of molds, *Aspergillus* was the most common (n = 10; 42%) followed by *Fusarium* (n = 4; 17%) and members of Mucorales (n = 4; 17%). Of yeasts, *C. tropicalis* was the most common (n = 6; 33%) followed by *C. albicans* (n = 5; 28%).

Comparing patients with any IFD to those with no IFD, demographics, and presence of complex cytogenetics and *FLT3*-ITD were similar. Significantly more patients with structural lung disease (p = 0.01), pre-chemotherapy neutropenia (p = 0.02), and *TP53* mutation (p = 0.04) developed IFD. There was a significant difference in the distribution of both clinical (p = 0.02) and molecular (p = 0.02) ontogeny between those who did and did not develop IFD.

**Conclusion:**

In absence of antifungal prophylaxis, structural lung disease, pre-chemotherapy neutropenia, and presence of a *TP53* mutation were enriched in patients who developed IFD within 100 days of receiving intensive induction chemotherapy for MM. This unbiased assessment of IFD risk factors suggests a targeted approach to antifungal prophylaxis in newly diagnosed MM patients may be reasonable to deploy.

**Disclosures:**

Jessica S. Little, MD, Merck and Company, Inc.: Grant/Research Support|Moderna, Inc.: Grant/Research Support

